# Modulation of jejunal mucosa-associated microbiota in relation to intestinal health and nutrient digestibility in pigs by supplementation of β-glucanase to corn–soybean meal-based diets with xylanase

**DOI:** 10.1093/jas/skab190

**Published:** 2021-06-14

**Authors:** Marcos Elias Duarte, Chris Sparks, Sung Woo Kim

**Affiliations:** 1 Department of Animal Science, North Carolina State University, Raleigh, NC 27695, USA; 2 Huvepharma, Inc., Peachtree City, GA, 30269, USA

**Keywords:** β-glucanase, growth performance, intestinal health, microbiota, nursery pigs, xylanase

## Abstract

This study aimed to evaluate the effects of increasing levels of β-glucanase on the modulation of jejunal mucosa-associated microbiota in relation to nutrient digestibility and intestinal health of pigs fed diets with 30% corn distiller’s dried grains with solubles and xylanase. Forty pigs at 12.4 ± 0.5 kg body weight (**BW**) were allotted in a randomized complete block design with initial BW and sex as blocks. Dietary treatments consisted of a basal diet with xylanase (1,500 endo-pentosanase units [**EPU**]/kg) and increasing levels of β-glucanase (0, 200, 400, and 600 U/kg) meeting nutrient requirements and fed to pigs for 21 d. Blood samples were collected on day 19. On day 21, all pigs were euthanized to collect intestinal tissues and digesta. Tumor necrosis factor-alpha, interleukin (**IL**)-6, and malondialdehyde were measured in the plasma and mid-jejunal mucosa. Viscosity was determined using digesta from the distal jejunum. Ileal and rectal digesta were evaluated to determine apparent ileal digestibility (**AID**) and apparent total tract digestibility (**ATTD**) of nutrients. Mucosa samples from the mid-jejunum were utilized for microbiota sequencing. Data were analyzed using the MIXED procedure on SAS 9.4. Overall, increasing dietary β-glucanase tended to increase (linear; *P* = 0.077) the average daily gain of pigs. Increasing dietary β-glucanase affected (quadratic; *P* < 0.05) the relative abundance of Bacteroidetes, reduced (linear; *P* < 0.05) *Helicobacter rappini*, and increased (linear, *P* < 0.05) *Faecalibacterium prausnitzii*. β-Glucanase supplementation (0 vs. others) tended to increase (*P* = 0.096) the AID of crude protein in the diet, whereas increasing dietary β-glucanase tended to increase (linear; *P* = 0.097) the ATTD of gross energy in the diet and increased (linear; *P* < 0.05) the concentration of IL-6 in the plasma of pigs. In conclusion, increasing β-glucanase up to 600 U/kg feed in a diet containing xylanase (1,500 EPU/kg) modulated mucosa-associated microbiota by increasing the relative abundance of beneficial bacteria and reducing potentially harmful bacteria. Furthermore, increasing β-glucanase up to 600 U/kg feed in a diet containing xylanase (1,500 EPU/kg feed) enhanced the status of the intestinal environment and nutrient utilization, as well as reduced systemic inflammation of pigs, collectively resulting in moderate improvement of growth performance. Supplementing β-glucanase at a range of 312 to 410 U/kg with xylanase at 1,500 EPU/kg feed showed the most benefit on jejunal mucosa-associated microbiota and reduced systemic inflammation of pigs.

## Introduction

Typical feeds for pigs are mainly composed of plant-based feedstuffs. Non-starch polysaccharides (**NSP**) present in plant-based feedstuffs are considered anti-nutritional factors due to their associated physicochemical properties. A typical corn and soybean meal-based diet contains about 2.3% to 3.8% of arabinoxylans (Knudsen, 1997; Jaworski et al., 2015) and 0.6% to 2.1% of β-glucans (Mathlouthi et al., 2002a; Sampson et al., 2015; Yu et al., 2018), whereas NSP concentrations are influenced by feedstuffs used in feeds. The use of coproducts such as distiller’s dried grains with solubles (**DDGS**) in feeds further increases NSP concentrations (Pedersen et al., 2014; Jaworski et al., 2015).

Major anti-nutritional roles of xylans and β-glucans are related to their capacity to increase digesta viscosity causing encapsulation of nutrients, thus reducing nutrient digestibility in feeds. In addition, increased digesta viscosity causes reduced passage rate, and thus increased amounts of undigested nutrients provide luminal environment for the proliferation of harmful microbiota (McDonald et al., 2001; Wellock et al., 2008; Metzler-Zebeli et al., 2010; Agyekum and Nyachoti, 2017) resulting in intestinal immune response, oxidative stress, and eventually reduced growth performance (Chen et al., 2020; Duarte et al., 2020; Kim and Duarte, 2021). These effects can be even more pronounced in nursery pigs due to their inability to adapt to dietary challenges as well as their limited capability of handling NSP (Lindberg, 2014; Niu et al., 2015).

In pig production, xylanase has been largely adopted to handle anti-nutritional effects of xylans, and the use of β-glucanase is increasing to handle β-glucans from grains (Adeola and Cowieson, 2011). The main mode of action is by reducing digesta viscosity which will, in turn, modulate the intestinal microbiota (Owusu-Asiedu et al., 2006; Zhang et al., 2018; Petry et al., 2020), increase nutrient digestibility, enhance the intestinal health, and finally improve growth performance of pigs (Ji et al., 2008; Passos et al., 2015; Chen et al., 2020). Most of the studies have evaluated supplemental effects of xylanase or β-glucanase individually (Li et al., 1996; Passos et al., 2015), whereas these enzymes are often supplemented together in pig production considering the complexity and the variety of NSP in plant-based feeds (Mathlouthi et al., 2002b; Tsai et al., 2017).

A recent study (Chen et al., 2020) evaluated the effects of increasing levels of xylanase on intestinal health and growth performance of nursery pigs. Chen et al. (2020) demonstrated that xylanase effectively enhanced intestinal health and growth performance of nursery pigs which were obtained at 1,500 endo-pentosanase units (**EPU**) xylanase/kg. Based on Chen et al. (2020), this study further evaluated the effects of increasing levels of β-glucanase in feeds containing 1,500 EPU xylanase/kg. Therefore, it was hypothesized that β-glucanase would hydrolyze β-glucan reducing digesta viscosity, increasing the digestibility of nutrients, increasing proliferation of health benefiting microbiota in the jejunal mucosa, and thus enhancing the intestinal health and growth of nursery pigs fed diets with xylanase. Thus, this study aimed to evaluate the effects of increasing levels of β-glucanase on the modulation of jejunal mucosa-associated microbiota in relation to nutrient digestibility and intestinal health of pigs fed corn–soybean meal-based diets supplemented with 30% corn DDGS and xylanase.

## Materials and Methods

The experimental procedures used in this study were reviewed and approved by the Institutional Animal Care and Use Committee at North Carolina State University.

### Animals, experimental design, and diets

Forty nursery pigs (20 barrows and 20 gilts) at 6 wk of age (21 d postweaning) and 12.4 ± 0.5 kg body weight (**BW**) were allotted to four dietary treatments (*n* = 10) based on a randomized complete block design with sex and initial BW serving as the blocks. A basal diet (Table 1) was formulated to meet the nutritional requirements suggested by NRC (2012). The experimental diets were supplemented with increasing levels of β-glucanase (0, 200, 400, and 600 U β-glucanase/kg feed) and xylanase (1,500 EPU/kg feed; Hostazym X 100, Huvepharma, Peachtree City, GA). The enzymes were premixed for each treatment to have an equal inclusion level of 0.01% for all treatments. Before the study, from weaning to day 21 postweaning, all pigs were fed a common corn–soybean meal-based diet formulated to meet the nutritional requirements suggested by National Research Council (NRC, 2012).

**Table 1.** Composition of experimental diets^1^ (as-fed basis)

Ingredient, %	Basal diet
Corn grain	39.28
Soybean meal	26.00
Corn DDGS	30.01
Poultry fat	2.00
l-Lys HCl	0.40
dl-Met	0.02
l-Thr	0.02
Salt	0.22
Vitamin premix^2^	0.03
Mineral premix^3^	0.15
Dical phosphate	0.61
Limestone	1.25
Xylanase + β-glucanase^1^	0.01
Phytase^4^	0.03
Total	100.00
Calculated composition	
DM, %	89.60
ME, Mcal/kg	3.40
SID^5^ Lys, %	1.24
Arabinoxylan^6^, %	6.92
β-Glucan^7^, %	1.95
Ca, %	0.72
STTD^8^ P, %	0.33
Total P, %	0.58
Analyzed composition	
DM, %	90.91
CP, %	21.14
Crude fat, %	5.53
Neutral detergent fiber, %	9.49
Acid detergent fiber, %	3.77

^1^Dietary treatments: β-glucanase providing 0, 200, 400, or 600 U/kg of feed. Mixtures of Hostazym X 100 (Huvepharma, Peachtree City, GA) to provide 1,500 EPU xylanase/kg of feeds for all treatments and β-glucanase to provide 0, 200, 400, and 600 U of β-glucanase/kg of feed for each treatment were premixed to have an equal inclusion level of 0.01% for all treatments. The analyzed enzyme activities in the premixes supplemented to experimental diets were: premix 1 (xylanase: 19,600 EPU/g), premix 2 (xylanase: 18,900 EPU/g and β-glucanase: 2,300 U/g), premix 3 (xylanase: 18,100 EPU/g and β-glucanase: 3,700 U/g); and premix 4 (xylanase: 18,500 EPU/g and β-glucanase: 6,700 U/g).

^2^The vitamin premix provided the following per kilogram of complete diet: 6,613.8 IU of vitamin A as vitamin A acetate, 992.0 IU of vitamin D_3_, 19.8 IU of vitamin E, 2.64 mg of vitamin K as menadione sodium bisulfate, 0.03 mg of vitamin B_12_, 4.63 mg of riboflavin, 18.52 mg of d-pantothenic acid as calcium pantothenate, 24.96 mg of niacin, and 0.07 mg of biotin.

^3^The trace mineral premix provided the following per kilogram of complete diet: 4.0 mg of Mn as manganous oxide, 165 mg of Fe as ferrous sulfate, 165 mg of Zn as zinc sulfate, 16.5 mg of Cu as copper sulfate, 0.30 mg of I as ethylenediamine di-hydroiodide, and 0.30 mg of Se as sodium selenite.

^4^OptiPhos 2000 was used as phytase (Huvepharma) at 0.025% to provide 500 FTU/kg feed.

^5^SID, standardized ileal digestible.

^6^Arabinoxylan content calculated according to Jaworski et al. (2015) and Tiwari et al. (2018).

^7^β-Glucan content calculated according to Sampson et al. (2015) and Yu et al. (2018).

^8^STTD P, standardized total tract digestible phosphorus.

The analyzed enzyme activity in the tested mixtures is described in Table 1. One EPU is the amount of enzyme needed for the release of 8.3 nmol of reducing sugars (xylose equivalent) per minute from oat spelt xylan at pH 4.7 and 50 °C. One unit of endo-1,4-β-glucanase (U) activity is defined as the amount of enzyme needed for the release of 0.128 micromoles of reducing sugars (glucose equivalents) per minute from barley β-glucan at pH 4.5 and 30 °C. 

Pigs were individually housed in pens and fed the assigned experimental diets for 21 d. BW and feed intake were measured weekly to analyze the average daily gain (**ADG**), average daily feed intake (**ADFI**), and gain to feed ratio (**G:F**) ratio as indicators of growth performance. On day 16 of the study, titanium dioxide (0.4%) was added to the feed as an indigestible marker to calculate the digestibility upon collection of digesta at day 21 of experiment.

### Samples collection and processing

Blood samples were collected on day 19 of the study into vacutainer tubes containing ethylenediaminetetraacetic acid (EDTA), placed on ice, and centrifuged (3,000 × *g* at 4 °C for 15 min) to obtain plasma. Samples were aliquoted and stored at −80 °C for further analysis of tumor necrosis factor-alpha (**TNF**-α), interleukin-6 (**IL**-**6**), and malondialdehyde (**MDA**) as indicators of systemic inflammatory status.

At the end of the study (day 21), all pigs were euthanized to collect intestinal tissues and digesta. Tissues from the mid-jejunum (3 m after the pyloric duodenal junction) and mid-colon were collected and washed with a sterile saline solution (0.9%). Each intestinal segment was longitudinally opened and gently scraped to obtain mucosa layers. The mucosa samples were collected into 2 mL vials, snap-frozen in liquid nitrogen, and then transferred to a −80 °C freezer. Intestinal mucosa samples collected from each segment (500 mg) were suspended in 1 mL of phosphate-buffered saline (**PBS**) and homogenized on ice with a tissue grinder (Tissuemiser; Thermo Fisher Scientific Inc. Waltham, MA). The homogenized mucosa samples were centrifuged at 14,000 × *g* at 4 °C for 3 min, and the supernatant was aliquoted into 0.5 mL vials and stored at −80 °C for further analysis of total protein, TNF-α, IL-6, and MDA. Section of the jejunum of each pig was fixed in 10% buffered formalin and further used for immunohistochemistry with Ki-67 staining and histomorphology measurement.

Digesta from the distal jejunum was collected into 50 mL tubes and placed on ice to measure viscosity immediately following collection. Digesta collected from the ileum and rectum (50 mL) were weighed, placed on ice, and then stored at −20 °C. In order to collect the sufficient volume of ileal digesta (50 mL), pigs were fasted for 6 h and refed for 6 h before euthanasia. The sample collection of all pigs was concluded within 2 h. The samples from the ileum and rectum were then freeze-dried, weighed, and finely ground for chemical analysis to calculate the digestibility of dry matter (**DM**), ether extract (**EE**), gross energy (**GE**), and crude protein (**CP**, 6.25 × N).

### Viscosity

Digesta collected from the distal jejunum were divided into two tubes (15 mL) and centrifuged at 1,000 × *g* at 4 °C for 10 min. The supernatant of each tube was collected into 2 mL tubes and centrifuged at 10,000 × *g* at 4 °C for 10 min. The supernatant was then transferred to a new 2-mL tube and kept on ice for the measurement of viscosity. A viscometer (Brookfield Digital Viscometer, Model DV-II Version 2.0, Brookfield Engineering Laboratories Inc., Stoughton, MA) was then used to measure the digesta viscosity at 25 °C. The viscosity was calculated as the average between 45.0/s and 22.5/s shear rates and recorded as millipascal-seconds (mPa.s) (Duarte et al., 2019; Chen et al., 2020).

### Microbiome sequencing

Mucosa samples collected from the mid-jejunum were used for mucosa-associated microbiome sequencing using the 16S rRNA gene sequence analysis. The DNA was extracted using the DNA Stool Mini Kit (#51604, Qiagen; Germantown, MD) following the instructions of the manufacturer. The extracted DNA samples were sent to the Genomics Department of Mako Medical Laboratories (Raleigh, NC) for 16S rRNA gene sequencing as reported by Jang et al. (2021). Briefly, extracted DNA samples were prepared for template on the Ion Chef instrument and sequencing on the Ion S5 system (Thermo Fisher Scientific Inc.). Variable regions V2, V3, V4, V6, V7, V8, and V9 of the 16S rRNA gene were amplified using Ion 16S Metagenomics Kit (Thermo Fisher Scientific Inc.). Sequences (Hypervariable regions) were processed using Torrent Suite Software (version 5.2.2; Thermo Fisher Scientific Inc.) to produce .bam files for further analysis. The taxonomy was assigned against the GreenGenes (anybody) and MicroSeq (experts) databases, specific primers for microbiota. Alpha and beta diversity rare fraction plot generation and the operational taxonomic unit (**OTU**) table generation were performed by the Ion Reporter Software Suite (version 5.2.2) of bioinformatics analysis tools (Thermo Fisher Scientific Inc.) with 98% similarity. The Ion Reporter’s Metagenomics 16S workflow powered by Qiime (version w1.1) was used to analyze the samples. The depth of sequencing coverage was > 1,000 × sample preparation. To initiate the statistical analysis of the microbiota, OTU data were transformed to relative abundance. The OTU with the relative abundance < 0.5% within each level was combined as “Others” (Holanda and Kim, 2020).

### Measurement of immune and oxidative stress status

Total protein, TNF-α, IL-6, and MDA concentrations were measured using colorimetric methods. A plate reader (Synergy HT, BioTek Instruments, Winooski, VT) and the software (Gen5 Data Analysis Software, BioTek Instruments) were used to measure the absorbance. The standard curves generated from the concentration and absorbance of the standard from each kit were used to calculate the respective concentration.

Total protein concentration in the mucosa of the mid-jejunum and mid-colon was determined using a bicinchoninic acid (**BCA**) Protein Assay (23225#, Thermo Fisher Scientific Inc.) as described by Chen et al. (2020). Prior to the assay, mucosa samples were tested to determine the dilution factor. Samples obtained from the mid-jejunum and mid-colon were diluted at (1:40) in PBS to enable the working range of the assay to be between 20 and 2,000 μg/mL. The wavelength to measure the absorbance was set at 562 nm. The total protein concentration was used to normalize the concentrations of TNF-α, IL-6, and MDA in the mucosa of mid-jejunum and mid-colon.

The concentration of TNF-α was measured in the plasma and mucosa of both the mid-jejunum and mid-colon using the Porcine TNF-α Quantikine ELISA Kit (PTA00; R&D System Inc. Minneapolis, MN) with a working range of 23.4 to 1,500 pg/mL as described by Jang et al. (2020). The concentration of IL-6 in the mucosa of jejunum was determined using the Porcine IL-6 Quantikine ELISA Kit (P6000B; R&D System Inc.) with a working range of 18.8 to 1,200 pg/mL as described by Duarte et al. (2020). Absorbance was measured at 450 and 540 nm, and the concentration of TNF-α and IL-6 was reported as pg/mL and pg/mg protein, respectively, in the plasma and mucosa.

The concentration of MDA in plasma and mucosa of the mid-jejunum and mid-colon was measured using the Thiobarbituric Acid Reactive Substance Assay Kit (STA-330, Cell Biolabs, San Diego, CA) with a working range of 0.98 to 125 μmol/L. The absorbance was measured at 532 nm, and the concentration of MDA in plasma and mucosa was reported as μmol/mL and μmol/mg protein, respectively (Zhao and Kim, 2020).

### Intestinal histomorphology and immunohistochemistry

Two sections of mid-jejunum per pig were cut placed in cassettes and sent to the North Carolina State University Histology Laboratory (College of Veterinary Medicine, Raleigh, NC) for staining using Ki-67 immunohistochemistry assay as previously reported by Holanda et al. (2020). Pictures of 10 well-oriented villi were taken at magnification 40× using a microscope Olympus CX31 (Lumenera Corporation, Ottawa, ON, Canada) with a camera Infinity 2-2 digital charge-coupled device (CCD) to measure the villus height (**VH**), villus width, and crypt depth (**CD**) and then calculate the VH to CD (**VH:CD**) ratio following Kim et al. (2019). Images of 10 intact crypts from each slide, taken at magnification 100×, were cropped and used to determine the enterocyte proliferation rate by analyzing the percentage of Ki-67-positive cells using the ImageJS software (Jang and Kim, 2019). All analyses of the intestinal histomorphology were executed by the same person. The averages of the 10 measurements per pig were used one unit for statistical analysis.

### Digestibility

Samples of diets and freeze-dried digesta from the ileum and rectum were analyzed to determine DM (method 934.01; AOAC, 2006), EE (method 2003.06; AOAC, 2006), GE using a calorimeter (6200, Parr Instrument Company, Moline, IL), CP using a TruSpec N Nitrogen Determinator (LECO Corp., St. Joseph, MI), and titanium dioxide following Myers et al. (2004). The digestibility of DM, GE, EE, and CP was calculated using the equation previously described by Duarte et al. (2019):

$$\begin{array}{l} Digestibility=\left\{1-\left[\left(TiO_{2diet}/TiO_{2digesta}\right)\times \left(Nutrient_{digesta}/Nutrient_{diet}\right)\right]\right\}\times 100; \end{array}$$

With TiO_2diet_ and TiO_2digesta_ denoting the concentrations of titanium dioxide in the diet and in the digesta from either the ileum or rectal; Nutrient_digesta_ and Nutrient_diet_ indicating the concentrations of nutrients in the digesta from the ileum or rectum as well as in the diet. The digestibility of nutrients was reported as apparent ileal digestibility (**AID**) and apparent total tract digestibility (**ATTD**).

### Statistical analyses

Data were analyzed using the MIXED procedure in SAS 9.4 (SAS Inc., Cary, NC). Dietary treatments were considered fixed effects. Initial BW blocks and sex were considered the random effect. The linear and quadratic effects of increasing levels of β-glucanase were tested by polynomial contrasts (*N* = 40). A preplanned contrast was performed using the CONTRAST statement to evaluate the effects of β-glucanase supplementation (0 vs. others). The coefficients for orthogonal polynomials were generated using the procedure IML. When a quadratic effect was found, the procedure RSREG was used to predict the critical value and the stationary point. The procedure CORR was used to generate the correlation among variables. Statistical differences were considered significant at *P* < 0.05. Tendency was considered at 0.05 ≤ *P* < 0.10.

## Results

### Growth performance

Increasing levels of β-glucanase did not affect the ADG of pigs until day 14 of experiment (Table 2), whereas it tended to increase ADG (linear; *P* = 0.068) of pigs during day 14 to 21 of the experiment. Overall, increasing levels of β-glucanase tended to increase (linear; *P* = 0.077) ADG of pigs without affecting ADFI or G:F during the 21 d period.

**Table 2.** Growth performance of pigs fed diet with β-glucanase

	β-Glucanase, U/kg feed^1^					*P-*value		
Item	0.0	200	400	600	SEM	Linear	Quadratic	0 vs. others
BW, kg								
Initial	12.4	12.4	12.4	12.3	0.5	0.990	0.925	0.983
Day 7	15.2	14.9	15.7	15.3	0.7	0.578	0.712	0.863
Day 14	20.0	19.1	20.9	20.2	1.0	0.247	0.824	0.933
Day 21	25.2	24.6	27.0	25.8	1.1	0.091	0.663	0.378
ADG, g/d								
Day 0 to 7	408	365	465	413	38	0.530	0.713	0.837
Day 7 to 14	682	602	742	700	55	0.275	0.632	0.991
Day 14 to 21	747	778	873	810	35	0.068	0.171	0.067
Overall	612	582	693	641	33	0.077	0.658	0.348
ADFI, g/d								
Day 0 to 7	577	535	633	573	41	0.415	0.708	0.898
Day 7 to 14	880	870	1,001	938	66	0.127	0.546	0.271
Day 14 to 21	1,452	1,384	1,605	1,526	84	0.119	0.925	0.461
Overall	970	930	1,080	1,013	61	0.118	0.760	0.404
G:F								
Day 0 to 7	0.71	0.68	0.72	0.72	0.03	0.627	0.745	0.981
Day 7 to 14	0.78	0.68	0.75	0.75	0.04	0.960	0.231	0.282
Day 14 to 21	0.52	0.57	0.55	0.54	0.03	0.707	0.261	0.265
Overall	0.63	0.62	0.65	0.64	0.02	0.537	0.957	0.841

^1^Four supplemental levels of β-glucanase (*N* = 40 total, *n* = 10 per supplemental level).

### Jejunal mucosa-associated microbiota

The average of sequence reads per sample was 14,084 ± 2,110. At the phylum level (Table 3), increasing levels of β-glucanase in the diet of nursery pigs did not affect the relative abundance of Proteobacteria, Firmicutes, Tenericutes, Spirochaetes, and Others (combined phyla with relative abundance lower than 0.05%). Increasing levels of β-glucanase, however, tended to increase (linear; *P* = 0.076) and affect (quadratic; *P* < 0.05) the relative abundance of Bacteroidetes (maximum 22.29% at 408 U/kg feed) in jejunal mucosa of nursery pigs. The Firmicutes to Bacteroidetes ratio tended to be affected (quadratic; *P* = 0.076) by increasing levels of β-glucanase in the diet.

**Table 3.** Relative abundance of jejunal mucosa-associated microbiota at the phylum level in nursery pigs fed diets with β-glucanase

	β-Glucanase, U/kg feed^1^					*P*-value		
Items	0.0	200	400	600	SEM	Linear	Quadratic	0 vs. others
Proteobacteria	35.08	39.80	19.66	33.22	9.67	0.535	0.629	0.689
Firmicutes	33.12	34.26	17.77	37.93	9.94	0.962	0.328	0.776
Tenericutes	25.72	22.53	31.88	17.33	11.75	0.768	0.632	0.898
Bacteroidetes	8.89	5.83	36.73	11.83	5.03	0.077	0.029	0.108
Spirochaetes	9.67	5.14	2.03	3.99	3.09	0.237	0.359	0.174
Others	0.06	0.14	0.04	0.07	0.06	0.822	0.655	0.675
F:B ratio^2^	6.23	16.07	0.42	8.04	3.83	0.246	0.097	0.439

^1^Four supplemental levels of β-glucanase (*N* = 40 total, *n* = 10 per supplemental level).

^2^F:B = Firmicutes/Bacteroidetes.

At the family level (Table 4), increasing levels of β-glucanase in the diet of nursery pigs affected (quadratic; *P* < 0.05) the relative abundance of Prevotellaceae (maximum 18.77% at 405 U/kg feed). Dietary inclusion of β-glucanase (0 vs. others) reduced the relative abundance of Campylobacteraceae (*P* < 0.05), whereas increasing levels of β-glucanase supplementation tended to reduce the relative abundance (linear; *P* = 0.084). Conversely, increasing levels of β-glucanase in the diet of nursery pigs increased (linear; *P* < 0.05) the relative abundance of Lachnospiraceae and Ruminococcaceae. Additionally, increasing levels of β-glucanase in the diet of nursery pigs affected (quadratic; *P* < 0.05) the relative abundance of others (maximum 4.70% at 382 U/kg feed), whereas β-glucanase supplementation tended to increase (*P* = 0.051) the abundance when compared with the diet without β-glucanase supplementation (0 vs. others).

**Table 4.** Relative abundance of jejunal mucosa-associated microbiota at the family level in nursery pigs fed diets with β-glucanase

	β-Glucanase, U/kg feed^1^					*P*-value		
Items	0.0	200	400	600	SEM	Linear	Quadratic	0 vs. others
*Mycoplasmataceae*	21.03	18.44	31.83	16.83	10.78	0.890	0.514	0.911
*Helicobacteraceae*	9.17	21.56	9.11	28.46	6.35	0.129	0.592	0.163
*Prevotellaceae*	6.75	5.26	28.65	10.39	4.81	0.261	0.040	0.133
*Lactobacillaceae*	7.20	12.63	6.95	21.21	6.78	0.200	0.552	0.424
*Streptococcaceae*	16.67	11.85	1.69	4.86	5.97	0.141	0.414	0.140
*Campylobacteraceae*	16.40	5.25	3.74	2.46	5.12	0.084	0.293	0.044
*Enterobacteriaceae*	8.02	10.50	1.74	0.04	5.41	0.225	0.817	0.537
*Veillonellaceae*	4.10	3.06	4.35	6.00	1.68	0.270	0.399	0.816
*Clostridiaceae*	4.49	4.50	0.60	2.06	2.66	0.433	0.706	0.503
*Brachyspiraceae*	3.87	1.54	1.01	1.32	1.39	0.234	0.316	0.122
*Moraxellaceae*	0.87	0.87	2.22	1.03	1.23	0.742	0.397	0.577
*Lachnospiraceae*	0.30	1.08	1.31	1.52	0.41	0.042	0.383	0.034
*Ruminococcaceae*	0.15	0.41	1.09	1.23	0.28	0.016	0.691	0.049
Others	0.99	3.03	5.65	2.92	1.58	0.230	0.044	0.051

^1^Four supplemental levels of β-glucanase (*N* = 40 total, *n* = 10 per supplemental level).

At the genus level (Table 5), increasing levels of β-glucanase in the diet of nursery pigs affected (quadratic; *P* < 0.05) the relative abundance of *Prevotella* (maximum 19.01% at 399 U/kg feed) and tended to reduce the relative abundance of *Streptococcus* (linear; *P* = 0.083) and *Campylobacter* (linear; *P* = 0.074). β-Glucanase supplementation reduced (*P* < 0.05) the relative abundance of *Campylobacter* when compared with the diet lacking β-glucanase (0 vs. others). Additionally, increasing levels of β-glucanase in the diet of nursery pigs increased (linear; *P* < 0.05) the relative abundance of *Faecalibacterium*. Increasing levels of β-glucanase in the diet of nursery pigs affected (quadratic; *P* < 0.05) the relative abundance of Others (maximum 5.35% at 380 U/kg feed), whereas β-glucanase supplementation tended to increase (*P* = 0.072) the abundance when compared with the diet lacking β-glucanase (0 vs. others).

**Table 5.** Relative abundance of jejunal mucosa-associated microbiota at the genus level in nursery pigs fed diets with β-glucanase

	β-Glucanase, U/kg feed^1^					*P*-value		
Items	0.0	200	400	600	SEM	Linear	Quadratic	0 vs. others
*Mycoplasma*	26.62	19.74	34.47	18.14	11.30	0.708	0.628	0.837
*Helicobacter*	13.94	26.85	11.02	31.15	7.54	0.203	0.616	0.296
*Lactobacillus*	11.04	13.82	7.61	23.00	7.40	0.301	0.407	0.655
*Prevotella*	6.75	4.86	29.35	9.92	4.93	0.296	0.039	0.150
*Streptococcus*	18.56	19.01	2.52	5.25	6.82	0.083	0.712	0.234
*Campylobacter*	11.60	4.36	1.86	1.28	3.78	0.074	0.321	0.048
*Sarcina*	5.57	1.18	0.17	2.05	2.89	0.542	0.163	0.189
*Selenomonas*	2.27	0.68	1.60	2.75	1.08	0.565	0.142	0.573
*Acinetobacter*	1.09	0.86	3.31	1.30	1.75	0.739	0.414	0.613
*Clostridium*	0.04	3.32	0.32	0.08	1.59	0.750	0.331	0.518
*Megasphaera*	1.02	0.76	0.89	1.12	0.43	0.793	0.551	0.827
*Faecalibacterium*	0.10	0.30	1.12	1.19	0.36	0.025	0.683	0.073
Others	1.39	4.27	5.75	3.16	1.50	0.392	0.049	0.072

^1^Four supplemental levels of β-glucanase (*N* = 40 total, *n* = 10 per supplemental level).

At the species level (Table 6), increasing levels of β-glucanase in the diet of nursery pigs affected (quadratic; *P* < 0.05) the relative abundance of *Prevotella copri* (maximum 22.71% at 410 U/kg feed) and *Prevotella_sp* (maximum 2.89% at 373 U/kg feed). Increasing levels of β-glucanase tended to reduce (linear; *P* = 0.094) the relative abundance of *Streptococcus alactolyticus*, reduced (linear; *P* < 0.05) the relative abundance of *Helicobacter rappini*, and increased (linear; *P* < 0.05) the relative abundance of *Faecalibacterium prausnitzii*. Additionally, increasing levels of β-glucanase tended to affect (quadratic; *P* = 0.056) the relative abundance of *Selenomonas bovis* (minimum 1.00% at 312 U/kg feed). The relative abundance of *Roseburia faecis* also tended to increase (*P* = 0.098) with dietary β-glucanase supplementation.

**Table 6.** Relative abundance of jejunal mucosa-associated microbiota at the species level in nursery pigs fed diets with β-glucanase

	β-gGucanase, U/kg feed^1^					*P-*value		
Items	0.0	200	400	600	SEM	Linear	Quadratic	0 vs. others
*Mycoplasma_sualvi*	24.03	18.64	31.02	22.28	11.22	0.967	0.834	0.997
*Prevotella_copri*	8.29	7.87	33.03	13.65	5.45	0.231	0.048	0.117
*Campylobacter_coli*	16.98	10.03	2.74	11.10	8.28	0.597	0.333	0.356
*Streptococcus_alactolyticus*	15.23	15.62	2.53	3.36	6.12	0.094	0.971	0.266
*Lactobacillus_mucosae*	3.56	2.62	5.02	9.17	3.45	0.226	0.538	0.615
*Streptococcus_hyointestinalis*	4.75	5.04	0.22	6.28	3.47	0.875	0.390	0.824
*Helicobacter_mastomyrinus*	4.43	5.87	1.04	3.51	2.44	0.607	0.743	0.737
*Helicobacter_rappini*	5.11	3.10	1.29	1.49	1.13	0.027	0.242	0.022
*Prevotella_stercorea*	1.11	0.73	3.39	1.80	0.94	0.368	0.380	0.396
*Prevotella_sp.*	0.87	0.31	4.90	0.88	1.09	0.571	0.042	0.284
*Selenomonas_bovis*	2.32	0.71	1.38	2.55	0.88	0.603	0.056	0.324
*Lactobacillus_delbrueckii*	0.18	4.54	0.23	1.85	2.11	0.845	0.580	0.416
*Acinetobacter_lwoffii*	1.04	1.70	3.56	1.94	2.03	0.482	0.259	0.292
*Helicobacter_equorum*	0.18	5.28	0.21	0.32	2.64	0.767	0.414	0.570
*Lactobacillus_kitasatonis*	0.27	2.57	0.17	0.88	1.30	0.888	0.237	0.330
*Sarcina_ventriculi*	2.52	1.00	0.10	1.84	1.45	0.746	0.251	0.362
*Lactobacillus_johnsonii*	1.86	0.75	0.18	1.85	1.06	0.996	0.151	0.399
*Faecalibacterium_prausnitzii*	0.17	0.85	1.55	1.96	0.52	0.015	0.621	0.033
*Selenomonas_lipolytica*	1.40	0.39	0.91	1.20	0.74	0.978	0.348	0.456
*Lactobacillus_sp.*	0.07	2.17	0.09	0.79	1.01	0.989	0.501	0.428
*Roseburia_faecis*	0.29	0.99	0.55	0.96	0.34	0.183	0.590	0.098
*Lactobacillus_vaginalis*	1.17	0.61	0.05	0.58	0.59	0.404	0.252	0.210
Others	4.08	8.55	4.29	2.26	1.82	0.257	0.118	0.652

^1^Four supplemental levels of β-glucanase (*N* = 40 total, *n* = 10 per supplemental level).

The alpha diversity of jejunal mucosa-associated microbiota estimated with Chao1 richness, Shannon diversity, and Simpson diversity was not affected by dietary β-glucanase supplementation (Table 7).

**Table 7.** α-Diversity of jejunal mucosa-associated microbiota estimated with Chao1 richness, Shannon diversity, and Simpson diversity

	β-Glucanase, U/kg feed^1^					*P*-value		
Items	0.0	200	400	600	SEM	Linear	Quadratic	0 vs. others
Family								
Chao1	11.60	14.50	17.4	16.06	3.30	0.156	0.396	0.130
Shannon	1.74	1.58	1.95	1.91	0.26	0.396	0.808	0.766
Simpson	0.55	0.49	0.58	0.58	0.07	0.556	0.638	0.982
Genus								
Chao1	9.81	12.60	13.80	14.06	2.27	0.121	0.517	0.106
Shannon	1.57	1.43	1.73	1.65	0.25	0.599	0.913	0.891
Simpson	0.52	0.46	0.54	0.50	0.07	0.884	0.848	0.801
Species								
Chao1	12.2	15.17	16.20	16.36	2.77	0.236	0.573	0.199
Shannon	1.95	2.06	1.98	2.06	0.33	0.883	0.967	0.941
Simpson	0.57	0.57	0.57	0.56	0.09	0.958	0.990	0.958

^1^Four supplemental levels of β-glucanase (*N* = 40 total, *n* = 10 per supplemental level).

### Immune and oxidative stress status

Increasing levels of β-glucanase supplementation did not affect the concentrations of TNF-α, IL-6, and MDA in the mucosa of the jejunum and colon (Table 8). However, increasing levels of β-glucanase in the diet of pigs tended to reduce (linear; *P* = 0.076) the concentration of TNF-α and reduced (linear; *P* < 0.05) the concentration of IL-6 in plasma. The concentration of MDA in plasma was not affected by dietary treatments.

**Table 8.** Immune parameters and oxidative stress status of pigs fed the diet with β-glucanase

	β-Glucanase, U/kg feed^1^					*P-*value		
Item	0.0	200	400	600	SEM	Linear	Quadratic	0 vs. others
Jejunum								
TNF-α, pg/mg protein	0.67	0.69	0.41	0.39	0.19	0.191	0.917	0.429
IL-6, pg/mg protein	2.60	2.73	2.71	2.74	0.27	0.710	0.848	0.654
MDA, µmol/g protein	0.55	0.61	0.51	0.57	0.08	0.820	0.954	0.938
Colon								
TNF-α, pg/mg protein	4.89	4.99	4.41	4.68	0.31	0.361	0.781	0.568
IL-6, pg/mg protein	3.08	3.20	3.11	2.92	0.24	0.577	0.488	0.987
MDA, µmol/g protein	1.11	1.11	1.18	0.96	0.12	0.359	0.244	0.806
Plasma								
TNF-α, pg/mL	108.1	93.4	99.2	89.3	6.6	0.076	0.700	0.056
IL-6, pg/mL	16.51	15.96	15.27	14.66	0.70	0.046	0.968	0.117
MDA, µmol/mL	8.41	8.62	8.56	8.02	1.05	0.776	0.709	0.990

^1^Four supplemental levels of β-glucanase (*N* = 40 total, *n* = 10 per supplemental level).

### Histomorphology, immunohistochemistry, and digesta viscosity

Increasing levels of β-glucanase increased the villus height (linear; *P* < 0.05) and tended to affect (quadratic; *P* = 0.073) the percentage of Ki-67-positive cells in the mid-jejunum of pigs (Table 9). Increasing levels of β-glucanase did not affect the villus width, crypt depth, and VH:CD ratio. Increasing levels of β-glucanase in the diets of nursery pigs did not affect the viscosity of digesta collected from the distal jejunum.

**Table 9.** Histomorphology and digesta viscosity in the jejunum of pigs fed the diet with β-glucanase

	β-Glucanase, U/kg feed^1^					*P-*value		
Item	0.0	200	400	600	SEM	Linear	Quadratic	0 vs. others
VH, µm	422	441	449	467	16	0.037	0.975	0.086
Villus width, µm	105	109	104	102	3	0.402	0.278	0.794
CD, µm	236	242	242	240	8	0.677	0.617	0.518
VH:CD ratio	1.80	1.83	1.86	1.95	0.08	0.141	0.741	0.311
Ki-67^+^, %	35.8	32.9	33.9	36.0	1.4	0.776	0.073	0.340
Viscosity, mPa·s	2.07	2.27	2.05	2.13	0.18	0.947	0.765	0.769

^1^Four supplemental levels of β-glucanase (*N* = 40 total, *n* = 10 per supplemental level).

### Digestibility

The increasing levels of β-glucanase did not affect the AID or ATTD of DM and EE in the diets of nursery pigs (Table 10). However, β-glucanase supplementation (0 vs. others) tended to increase (*P* = 0.096) the AID of CP in the diet of nursery pigs. In addition, increasing levels of β-glucanase tended to increase (linear; *P* = 0.097) the ATTD of GE in diet of nursery pigs.

**Table 10.** AID and ATTD of DM, CP, EE, and GE in diets with β-glucanase fed to pigs

	β-Glucanase, U/kg feed^1^					*P-*value		
Item	0.0	200	400	600	SEM	Linear	Quadratic	0 vs. others
AID, %								
DM	56.2	58.1	58.4	59.1	2.74	0.447	0.819	0.430
CP	67.3	72.8	69.3	71.8	2.54	0.289	0.481	0.096
EE	69.3	72.4	76.1	69.9	3.44	0.676	0.137	0.294
GE	61.3	63.3	63.5	63.4	2.55	0.541	0.674	0.437
ATTD, %								
DM	70.4	70.9	70.3	73.9	1.89	0.159	0.314	0.471
CP	71.1	73.4	71.7	75.3	1.91	0.123	0.659	0.186
EE	70.7	72.1	72.5	71.6	2.61	0.754	0.608	0.585
GE	68.9	70.3	69.6	73.1	1.93	0.097	0.485	0.246

^1^Four supplemental levels of β-glucanase (*N* = 40 total, *n* = 10 per supplemental level).

### Correlation with mucosa-associated microbiota

Mycoplasmataceae was positively correlated with the concentration of MDA in the colonic mucosa (*r* = 0.37; *P* < 0.05), whereas it was negatively correlated with the AID of DM (*r* = −0.38; *P* < 0.05) and GE (*r* = −0.35; *P* < 0.05) (Table 11). Similarly, *Mycoplasma sualvi* was negatively correlated with the AID of DM (*r* = −0.40; *P* < 0.05) and GE (*r* = −0.37; *P* < 0.05). Campylobacteraceae was negatively correlated with ADG (*r* = −0.33; *P* < 0.05). Similarly, *Campylobacter coli* was negatively correlated with the final BW (*r* = −0.42; *P* < 0.05), ADG (*r* = −0.46; *P* < 0.05), and ADFI (*r* = −0.43; *P* < 0.05). However, *C. coli* was positively correlated with AID of DM (*r* = 0.35; *P* < 0.05) and GE (*r* = 0.35; *P* < 0.05). Enterobacteriaceae was negatively correlated with the VH:CD in the jejunum (*r* = −0.33; *P* < 0.05). *Helicobacter mastomyrinus* was positively correlated with the concentration of MDA in the jejunal mucosa (*r* = 0.37; *P* < 0.05). *Helicobacter rappini* and *Helicobacter equorum* were positively correlated with the concentration of TNF-α in the jejunal mucosa (*r* = 0.50, *r* = 0.32; *P* < 0.05, respectively). *Lactobacillus delbrueckii* and *Lactobacillus_sp.* were positively correlated with the VH:CD in the jejunal mucosa (*r* = 0.40, *r* = 0.42; *P* < 0.05, respectively).

**Table 11.** Pearson correlation coefficients (*r*) between mucosa-associated microbiota and other variables measured in pigs fed diets with β-glucanase

Item	Family (*P*-value, *r*)	Species (*P-*value, *r*)		
BW	*Campylobacteraceae * (0.089, −0.28)	*Campylobacter_coli* (0.008, −0.42)		
ADG	*Campylobacteraceae * (0.040, −0.33)	*Campylobacter_coli* (0.004, −0.46)		
ADFI	*Campylobacteraceae * (0.078, −0.29)	*Campylobacter_coli* (0.006, −0.43)		
VH:CD ratio	*Enterobacteriaceae * (0.043, −0.33)	*Helicobacter_rappini* (0.021, −0.36)	*Lactobacillus_delbrueckii* (0.011, *r* = 0.40)	*Lactobacillus_sp.* (0.008, 0.42)
TNFa, jejunum	*Enterobacteriaceae * (0.001, 0.54)	*Helicobacter_rappini* (0.001, 0.50)	*Helicobacter_equorum* (0.049, *r* = 0.32)	
MDA, jejunum		*Helicobacter_mastomyrinus* (0.028, 0.37)		
AID, DM	*Mycoplasmataceae * (0.031, −0.38)	*Mycoplasma_sualvi* (0.022, −0.40)		
AID, GE	*Mycoplasmataceae * (0.049, −0.35)	*Mycoplasma_sualvi* (0.033, −0.37)		

## Discussion

In this study, increasing levels of β-glucanase within the corn–soybean meal-based diet supplemented with 30% corn DDGS in combination with xylanase showed a trend to improve the growth performance and effectively affect the intestinal microbiota reducing, therefore, the systemic inflammatory status of nursery pigs. The trend to improve the growth performance from day 14 to 21 of the experiment by increasing ADG occurred without an alteration in ADFI or feed efficiency. The use of dietary xylanase or β-glucanase in swine diets has been reported to be effective in reducing digesta viscosity, affecting the intestinal microbiota, and enhancing the intestinal health (Duarte et al., 2019; Chen et al., 2020; Petry et al., 2020). The improvement in the intestinal environment can further increase the digestibility of nutrients and consequently the growth performance of pigs (Ji et al., 2008; Duarte et al., 2019; Chen et al., 2020). Whereas these parameters have been shown to be further improved with the inclusion of β-glucanase combined with xylanase due to the variety of NSP present in plant-based diets (Mathlouthi et al., 2002b; Knudsen, 2014; Tsai et al., 2017). The composition and structure of cell wall in cereal grains vary depending on different parts in the grain (Dien et al., 2005; Yoshida et al., 2012). The concentration of xylan in the pericarp is greater than in the endosperm (Dien et al., 2005). In addition, the cellulose microfibrils in the cell wall are embedded in arabinoxylans, β-glucans, and structural proteins (Chandrashekar and Mazhar, 1999; Stone et al., 2010; Kiemle et al., 2014). Therefore, it can be suggested that feeding a blend of enzymes may be more effective in reducing the anti-nutritional effect of NSP in the diet of nursery pigs.

In the current study, pigs were fed a basal diet with an increased fiber content (9.5% neutral detergent fiber [NDF] and 3.8% acid detergent fiber [ADF]. It is well known that high-fiber diets can increase the digesta viscosity, reducing the digestibility of nutrients (Chen et al., 2020; Hung et al., 2021). The main functions of β-glucanase and xylanase are to hydrolyze the polymers β-glucan and xylans, respectively, therefore reducing their capacity to increase digesta viscosity and increasing their fermentability by the microbiota (Passos et al., 2015; Duarte et al., 2019; Chen et al., 2020; Petry et al., 2020). In addition, hydrolysis of NSP in the cell wall can release nutrients encapsulated within the cell (Bedford and Partridge, 2000; Baker et al., 2021). The lower viscosity and released nutrients can increase the interaction of the enzymes and substrates resulting in increased digestion and absorption of nutrients. Increasing levels of β-glucanase in diets did not further reduce digesta viscosity in this study possibility because all diets contained xylanase at the same level. However, it showed a trend to increase the AID of CP and the ATTD of GE. These results indicate that enhancement of AID of CP could also be associated with the capacity of NSP-degrading enzymes to release nutrients from the cells in the small intestine. In addition, Fan et al. (2009) reported that β-glucanase and xylanase supplementation increased the activities of pepsin in gastric mucosa and γ- glutamyl transpeptidase and disaccharidases in jejunal and ileal mucosa of weaned pigs fed barley-based diet. The hydrolysis of NSP releases oligosaccharides, increasing the fermentability of the dietary fiber by microbes and production of short-chain fatty acids (**SCFA**) along the intestine (Lafond et al., 2012; Masey-O′Neill et al., 2014) that can then be rapidly absorbed by epithelial cells, effectively altering energy metabolism (den Besten et al., 2013; Nakatani et al., 2018). This process can partly explain the improvement in ATTD of GE seen in the present study.

The changes in the physicochemical properties of dietary fiber and the resulting nutrient availability caused by the use of NSP-degrading enzymes (Ji et al., 2008; Passos et al., 2015; Chen et al., 2020) alter the environment in the intestinal lumen, leading to a shift toward a more beneficial microbiota (Munyaka et al., 2016; Zhang et al., 2018; Akkerman et al., 2020). The change in the environment of the small intestine may partially explain the modulation of the mucosa-associated microbiota among treatments observed in this study. Adhikari et al. (2019) have demonstrated that the microbiota in the lumen of jejunum differ from those associated with the mucosa. The jejunum is the major site for digestion and absorption of amino acids (AA), energy, and fat with a considerable amount of fiber fermentation (Mace and Marshall, 2013; Passos et al., 2015; Tiwari et al., 2018; Chen et al., 2020). In addition to the digestive functions, the jejunum plays important role in the immune system. According to Wiarda et al. (2020), the immune cells are more abundant in the jejunum than in the large intestine in nursery pigs. According to Petry et al. (2020), the interaction among diet, microbiota, and immune system responses is more effective in the small intestine. In addition, it has been demonstrated that the mucosa-associated microbiota directly interact with intestinal immune cells (Mulder et al., 2011; Arpaia et al., 2013; Belkaid and Hand, 2014) leading to a greater capability on immunological regulation (Mu et al., 2017). Furthermore, the mucosa-associated microbiota is greatly susceptible to dietary influence in the small intestine of pigs (Levesque et al., 2012, 2014). Therefore, considering the digestive functions and the physicochemical properties in the small intestine, Duarte and Kim (2021) have showed that the mucosa of the jejunum could be considered a key site to evaluate the diet, intestinal microbiota, and intestinal health interaction. In this study, increasing levels of β-glucanase within diets containing xylanase reduced the abundance of the proteolytic bacteria *H. rappini* and increased the abundance of the fiber-degrading families *Prevotellaceae*, *Lachnospiraceae*, and *Ruminococcaceae*. Increasing the AID of CP results in a reduction of the availability of undigested protein for microbial fermentation, thus reducing the abundance of proteolytic bacteria. In addition, the oligosaccharides released by xylanase and β-glucanase can modulate the mucosa-associated microbiota by selectively increasing the abundance of fiber-degrading bacteria (Munyaka et al., 2016; Baker et al., 2021; Petry et al., 2021). A lower abundance of proteolytic bacteria such as *Helicobacter* spp. has been correlated with improved health status in pigs (Dowd et al., 2008; Duarte et al., 2020). Protein fermentation releases a broad range of metabolites within the gastrointestinal tract that can be harmful to the host by affecting the immune response and intestinal permeability (Richter et al., 2014; Villodre Tudela et al., 2015). Interestingly, fiber fermentation is more related to the production of SCFA by bacteria such as *Lachnospiraceae* and *Ruminococcaceae* (Venegas et al., 2019). In pigs fed plant-based diets, increasing abundance of the family Prevotellaceae, which belongs to the phylum Bacteroidetes, in the intestinal mucosa pigs has indicated enhanced healthy status of the host (Adhikari et al., 2019; Duarte et al., 2020).

The increase in the relative abundance of the family *Ruminococcaceae* might be attributed to the increase of the species *F. prausnitzii*, which has been identified as a potential probiotic for humans due to its benefits to the host, namely its anti-inflammatory properties (Martín et al., 2017). *Faecalibacterium prausnitzii* is a Gram-negative bacteria lacking lipopolysaccharides within its cell wall with a variety numbers of anti-inflammatory mechanisms (Miquel et al., 2013; Qiu et al., 2013; Zhang et al., 2021). *Faecalibacterium prausnitzii* directly interact with the epithelial cells by secreting metabolites that further affect the immune system (Zhang et al., 2021). Butyric acid is produced by *F. prausnitzii* (Ganesan et al., 2018), utilizing mainly acetic acid as substrate (Louis et al., 2007), and therefore its growth is generally attributed to acetic acid-producing bacteria such as *Lactobacillus* and *Bifidobacterium* (Miquel et al., 2014). Butyric acid has been shown to promote improved intestinal health in nursery pigs reducing the concentration of TNF-α and interferon gamma (IFN-γ) (Wen et al., 2012; Diao et al., 2019; Zhong et al., 2019). In addition, the metabolites produced by *F. prausnitzii* possess anti-inflammatory effects that are able to block nuclear factor kappa B (NF-κB) activation and the IL-8 production (Sokol et al., 2008; Miquel et al., 2015) by downregulating Toll-like receptors 3 and 4 (**TLR**3 and TLR4) in the intestinal mucosa (Zhang et al., 2021). Therefore, the supplementation of β-glucanase within the diet in combination with xylanase modulated the jejunal mucosa-associated microbiota leading to a reduction in the systemic inflammatory status of nursery pigs.

β-Glucans can directly affect the immune system by binding receptors in the intestine and increasing dectin-1 stimulation (Kanjan et al., 2017). Dectin-1 activates the immune response, effectively stimulating the production of pro-inflammatory cytokines (Sahasrabudhe et al., 2016; Kanjan et al., 2017). After enzymatic treatment of NSP-containing feed ingredients, these effects are even more pronounced (Sahasrabudhe et al., 2016), whereas β-glucans can affect the adherence of bacteria in the epithelial cells further affecting the immune response (Arena et al., 2016). In the present study, the systemic inflammatory status was linearly enhanced by reducing the concentration of IL-6 and TNF-α in the plasma, possibly due to the modulation of the microbiota. According to Kreuzer et al. (2012), metabolites produced by protein fermentation upregulate the expression of IL-6; therefore, a reduction of proteolytic bacteria may have contributed to the reduction of IL-6 seen in the present study. In addition, the shift in the microbiota could have affected the production of SCFA that have been shown to inhibit inflammation within the intestine of pigs (Wen et al., 2012). In light of this, evaluating the role of NSP-degrading enzymes on inflammatory status should also take into account the interaction between fiber fermentation and protein availability on the intestinal microbiota, considering the modulation in the microbiota composition (Pieper et al., 2012; Richter et al., 2014). Therefore, β-glucanase within the diet with xylanase can reduce the systemic inflammation of nursery pigs.

Although the increasing β-glucanase supplementation did not affect the immune and oxidative stress status on jejunal mucosa, the enhancement of the immune status parameters in serum may have reduced the epithelial damage by enhancing the status of the intestinal environment, reducing the deleterious effect of the dietary fiber, and consequently increasing the villus height on nursery pigs in this study. In addition, the greater villus height can be a consequence of a greater enterocyte proliferation rate (Wang et al., 2019; Zhong et al., 2019). Conversely, Duarte et al. (2020) reported that lower VH is correlated with greater cell proliferation rate. In this study, the enterocyte proliferation rate, indicated by the percent of Ki-67^+^ crypt cells, showed a quadratic effect with the increasing β-glucanase supplementation. This result indicates that the β-glucanase supplementation increased the cell proliferation by enhancing the status of the intestinal environment and increasing the abundance of butyric acid-producing bacteria (Zhong et al., 2019). According to Knudsen et al. (2012), some fatty acids in the lumen stimulate the enterocyte proliferation. The enhancement in the intestinal barrier function can also be attributed to the increased abundance of *F. prausnitzii* in the jejunal mucosa due to its anti-inflammatory effects (Zhang et al., 2021). Therefore, enhancing the status of the intestinal environment can be associated with the improvement of the intestinal integrity.

In this study, considerable changes in the microbiota by supplemental blend of enzymes led to a reduction in the systemic immune response and an enhancement in the intestinal structure, which in turn caused a tendency to increase the growth performance of pigs. The correlation test showed that the growth performance was negatively correlated with *Campylobacter coli*, which belongs to the family *Campylobacteraceae*. The VH:CD was negatively correlated with *Enterobacteriaceae* and *H. rappini*, whereas it was positively correlated with *Lactobacillus* spp. The concentration of TNF-α in the jejunum was positively correlated with *Enterobacteriaceae* and *Helicobacter* spp. The concentration of MDA in the jejunum was positively correlated with *H. mastomyrinus*. The AID of DM and GE was negatively correlated with *M. sualvi*. The bacteria correlated with growth performance, intestinal health, and digestibility related in this study were previously reported by Duarte and Kim (2021) as key microbiota. Considering that growth performance is a multifactorial variable, it can be suggested that the shift of the microbiota should affect other parameters first and then affect growth performance indirectly. As recently suggested by Petry et al. (2020), a longer adaptation period is needed for testing xylanase supplementation for pigs. The initial BW of pigs was 12.3 kg in this study; at this age, the microbiota has increased the ability to handle NSP within diets (Niu et al., 2015; Ke et al., 2019). This emphasizes the importance of further experiments to determine specific metabolites in the context of long-term dietary NSP-degrading enzyme supplementation, considering dietary fiber intake, age of pigs, the microbiota profile at the beginning of the study, as well as the duration of the study.

In conclusion, increasing β-glucanase up to 600 U/kg feed in a diet containing xylanase (1,500 EPU/kg feed) modulated mucosa-associated microbiota by increasing the relative abundance of beneficial bacteria and reducing potentially harmful bacteria. Furthermore, increasing β-glucanase up to 600 U/kg feed in a diet containing xylanase (1,500 EPU/kg feed) enhanced the status of intestinal environment and the nutrient utilization as well as reduced the systemic inflammation of pigs, collectively resulting in a moderate improvement of growth performance. Supplementing β-glucanase at a range of 312 to 410 U/kg feed with xylanase at 1,500 EPU/kg feed showed the most benefit on mucosa-associated microbiota in the jejunum and reduced systemic inflammation of pigs.

## Abbreviations

ADFI: average daily feed intake
ADG: average daily gain
AID: apparent ileal digestibility
ATTD: apparent total tract digestibility
BCA: bicinchoninic acid
BW: body weight
CP: crude protein
DDGS: distiller’s dried grains with solubles
DM: dry matter
EPU: endo-pentosanase units
F:B: Firmicutes/Bacteroidetes
FTU: phytase unit
G:F: gain to feed ratio
GE: gross energy
IL-6: interleukin-6
MDA: malondialdehyde
NSP: non-starch polysaccharide(s)
OTU: operational taxonomic unit
PBS: phosphate-buffered saline
SID: standardized ileal digestible
STTD: standardized total tract digestible
TLR3: Toll-like receptors 3
TLR4: Toll-like receptors 4
TNF-α: tumor necrosis factor-alpha
VH:CD ratio: villus height to crypt depth ratio
